# Identified a disintegrin and metalloproteinase with thrombospondin motifs 6 serve as a novel gastric cancer prognostic biomarker by bioinformatics analysis

**DOI:** 10.1042/BSR20204359

**Published:** 2021-04-22

**Authors:** Ya-zhen Zhu, Yi Liu, Xi-wen Liao, Shan-shan Luo

**Affiliations:** 1 Department of Gastrointestinal Surgery, Guangxi Medical University Affiliated Tumor Hospital, Guangxi Clinical Research Center for Colorectal Cancer, Nanning 530021, Guangxi Zhuang Autonomous Region, People’s Republic of China; 2 Department of Hepatobiliary Surgery, The First Affiliated Hospital of Guangxi Medical University, Nanning 530021, Guangxi Zhuang Autonomous Region, People’s Republic of China

**Keywords:** ADAMTS, gastric cancer, mRNA, prognosis

## Abstract

**Objective:** We aimed to explore the prognostic value of a disintegrin and metalloproteinase with thrombospondin motifs (*ADAMTS*) genes in gastric cancer (GC). **Methods:** The RNA-sequencing (RNA-seq) expression data for 351 GC patients and other relevant clinical data were acquired from The Cancer Genome Atlas (TCGA). Survival analysis and a genome-wide gene set enrichment analysis (GSEA) were performed to define the underlying molecular value of the *ADAMTS* genes in GC development. Besides, qRT-PCR and immunohistochemistry were all employed to validate the relationship between the expression of these genes and GC patient prognosis. **Results:** The Log rank test with both Cox regression and Kaplan–Meier survival analyses showed that *ADAMTS*6 expression profile correlated with the GC patients clinical outcome. Patients with a high expression of *ADAMTS*6 were associated with poor overall survival (OS). Comprehensive survival analysis of the *ADAMTS* genes suggests that *ADAMTS*6 might be an independent predictive factor for the OS in patients with GC. Besides, GSEA demonstrated that *ADAMTS*6 might be involved in multiple biological processes and pathways, such as the vascular endothelial growth factor A (VEGFA), kirsten rat sarcoma viral oncogene (KRAS), tumor protein P53, c-Jun N-terminal kinase (JNK), cadherin (CDH1) or tumor necrosis factor (TNF) pathways. It was also confirmed by immunohistochemistry and qRT-PCR that ADAMTS6 is highly expressed in GC, which may be related to the prognosis of GC patients. **Conclusion:** In summary, our study demonstrated that *ADAMTS*6 gene could be used as a potential molecular marker for GC prognosis.

## Introduction

Gastric cancer (GC) is the fourth most common cancer and the second cause of mortality worldwide [1]. The median survival time of patients with GC recurrence and metastasis is less than 1 year [2,3]. According to the 2015 data at the National Cancer Center, there were ∼679000 new stomach cancer cases and 498000 deaths [4]. China accounts for 430000 new GC cases and 300000 deaths every year [5]. The GC development may be a process of long-term synergistic action of multiple factors. GC might be triggered by pathogenic infections, such as *Helicobacter pylori* (HP) [6–9] or gastric ulcers [10], chronic atrophic gastritis [11], carcinogens such as nitrite in food [12], smoking [13,14], and long-term drinking [15]. Despite the immense progress made in the clinical management of GC, the prognosis and survival rates of patients remain poor. Low early diagnosis rate and high local recurrence cases coupled with distant metastasis rates for advanced GC results in the poor GC prognosis in China. It is, therefore, vital to further interrogate the carcinogenesis and development of GC in order to develop new prognostic molecular markers and targeted therapy.

A disintegrin and metalloproteinase with thrombospondin motifs (*ADAMTS*) genes are zinc-dependent metalloproteases. Previous research has demonstrated a close association between *ADAMTS* and tumor invasion and metastasis [16]. *ADAMTS* participate in multiple biological pathways, including histomorphogenesis, pathophysiological reconstruction, inflammation, angiogenesis, or tumorigenesis [17,18]. However, the diagnostic, prognostic, or therapeutic value of the *ADAMTS* genes in the development of GC is yet to be defined. Here, using The Cancer Genome Atlas (TCGA) and the Kaplan–Meier plotter (KM plotter) tools, we explored the diagnostic and underlying prognostic value of the *ADAMTS* family of genes in stomach cancer.

## Methods

### Public database source

The RNA-sequencing (RNA-seq) expression data for 383 patients and relevant clinical data were acquired from the TCGA database (https://portal.gdc.cancer.gov/; accessed 15 May 2019). The mined data comprised 351 GC tumors and 32 normal gastric samples. The raw data were normalized via the DESeq (https://www.bioconductor.org/packages/release/bioc/html/DESeq.html) [19].

### Bioinformatics analysis

We used GraphPad Prism 8 to draw the scatter diagram and receiver operating characteristic (ROC) curves of the expression distribution for both the GC and the normal samples. The unpaired *t* test was used to compare the differences shown in the scatter diagram and the area under ROC curve. Using the Database for Annotation, Visualization and Integrated Discovery (DAVID, (https://david.ncifcrf.gov/home.jsp; accessed 1 December 2019; v.6.8), we then investigated the Gene Ontology (GO) enrichment of *ADAMTS* genes [20]. We used the gene multiple association network integration algorithm (Gene MANIA; http://www.genemania.org/; accessed 1 December 2019) to construct the gene–gene networks [21,22] and the Search Tool for the Retrieval of Interacting Genes/Proteins (STRING v.10.0; https://string-db.org/; accessed 1 December 2019) to define the protein–protein interaction (PPI) networks [23,24].

### Comprehensive survival analysis of *ADAMTS* genes

Using the median values for the gene expression profile, GC patients were classified into two categories based on survival analysis. Both the *ADAMTS* expression and clinical data in GC tissues or adjacent tissues were analyzed using the log-rank test, while the clinical characteristics related to overall survival (OS) were selected and adjusted by the multivariate Cox regression survival analysis. Besides, the clinical pathological features were further analyzed in the subgroups. We then conducted full survival analysis using the prognosis-related *ADAMTS* genes, and assessed the survival ROC curves using the R package platform, as well as the total and subgroup survival analysis. Finally, the prognostic relationship between *ADAMTS* family of genes and GC was verified by the KM plotter database (www.kmplot.com).

### Gene set enrichment analysis

We used gene set enrichment analysis (GSEA) (http://software.broadinstitute.org/gsea/index.jsp; accessed 15 December 2020) [25,26] to study the biological differences and pathways affected by the differential expression of the *ADAMTS* gene pool and their relationship with GC clinical outcome. The GSEA software applies the Molecular Signatures Database (MSigDB) c2 (c2.cp.kegg.v7.0.symbols.gmt) and c5 (c5.all.v7.0.symbols.gmt) [27] in gene set analysis. Then a probability value of <0.05 and a false discovery rate (FDR) < 0.25 for GSEA were considered statistically significant.

### qRT-PCR

We collected 24 pairs of GC and adjacent normal tissue samples surgically from December 2020 to February 2021 at Guangxi Medical University Affiliated Tumor Hospital for RNA extraction. A cDNA reverse transcription kit (RR036A; TaKaRa, U.S.A.) and TB Green kit (RR820A; TaKaRa, U.S.A.) were used for reverse transcription into cDNA and qRT-PCR. Primers used were: ADAMTS6-F: 5′-CCTCCCAAGCGTGACTTTCT-3′; ADAMTS6-R: 5′-AGACACCAGAGCTCTCTACACACTT-3′. GAPDH-F: 5′-CAGGAGGCATTGCTGATGAT-3′, and GAPDH-R: 5′-GAAGGCTGGGGCTCATTT-3′.

### Immunohistochemistry

We collected 18 pairs of GC tissues and paired paracancerous tissue samples in the Guangxi Medical University Affiliated Tumor Hospital from January 2018 to December 2018. The experiments were approved by the Ethical Committee of the Guangxi Medical University Affiliated Tumor Hospital and written informed consent was signed by each participant. The sections were placed in an oven at 65°C for 2 h, dewaxed with xylene, hydrated with a graded series of ethanol, and repaired with antigen repair buffer using the EDTA method. Endogenous antigens were blocked with 3% hydrogen peroxide. The sections were incubated with Rabbit Anti-ADAMTS6 antibody (bs-8009R) overnight at 4°C. The sections were heated for 30 min to bring them to room temperature and incubated with secondary antibody for 20 min. Staining was visualized using DAB color developing solution. The sections were stained with Hematoxylin, differentiated in 1% hydrochloric acid in alcohol, and dehydrated. Thereafter, the sections were dried naturally in a fume hood, transparentized with xylene, and sealed with neutral resin. The negative control contained PBS instead of primary antibody, and a proven positive section served as the positive control. The staining index was scored according to staining intensity (0, no staining; 1, weak, light yellow; 2, moderate, yellow brown; 3, strong, brown) and the proportion of positive cells (0, 0%; 1, <10%; 2, <50%; 3, <75%; 4, >76%). An ‘immunoreactive score’ was determined to be the product of the intensity and percentage of positive cells, which ranged from 0 to 12. Cases with scores of 0–7 were defined as the negative group and those with scores of 8–12 were the positive group [28].

### Statistical analysis

We employed the Benjamini–Hochberg program to adjust FDRs in GSEA for multiple tests [29–31]. The statistical analysis was conducted via SPSS version 23.0 (IBM Corporation, Armonk, NY, U.S.A.). A probability value of *P*<0.05 was considered statistically significant.

## Results

**Figure 1:**
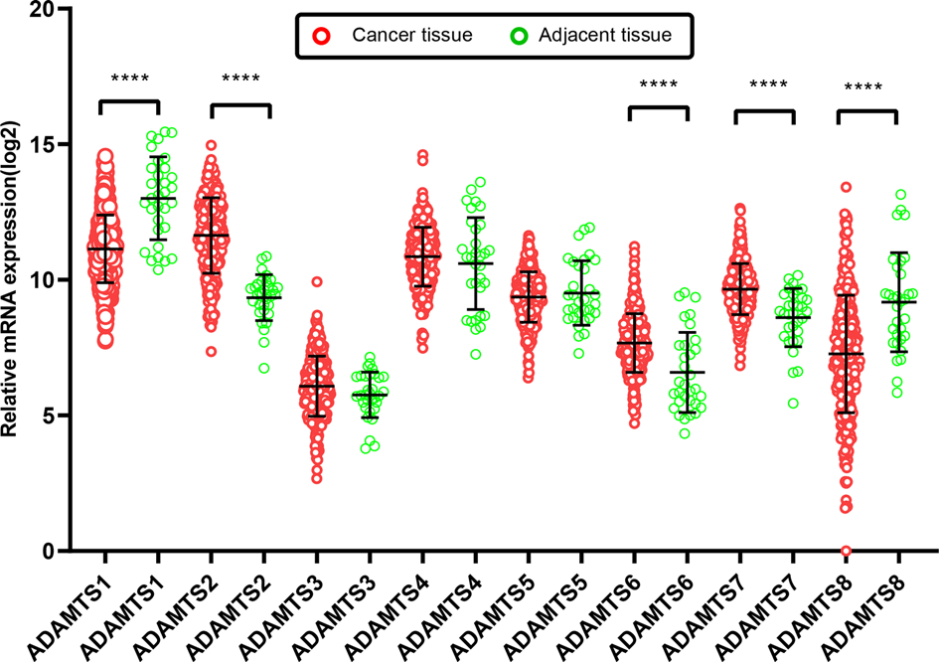
The scatter plot of *ADAMTS* mRNA expression in GC and adjacent tissues based on TCGA database ****P<0.0001.

**Figure 2:**
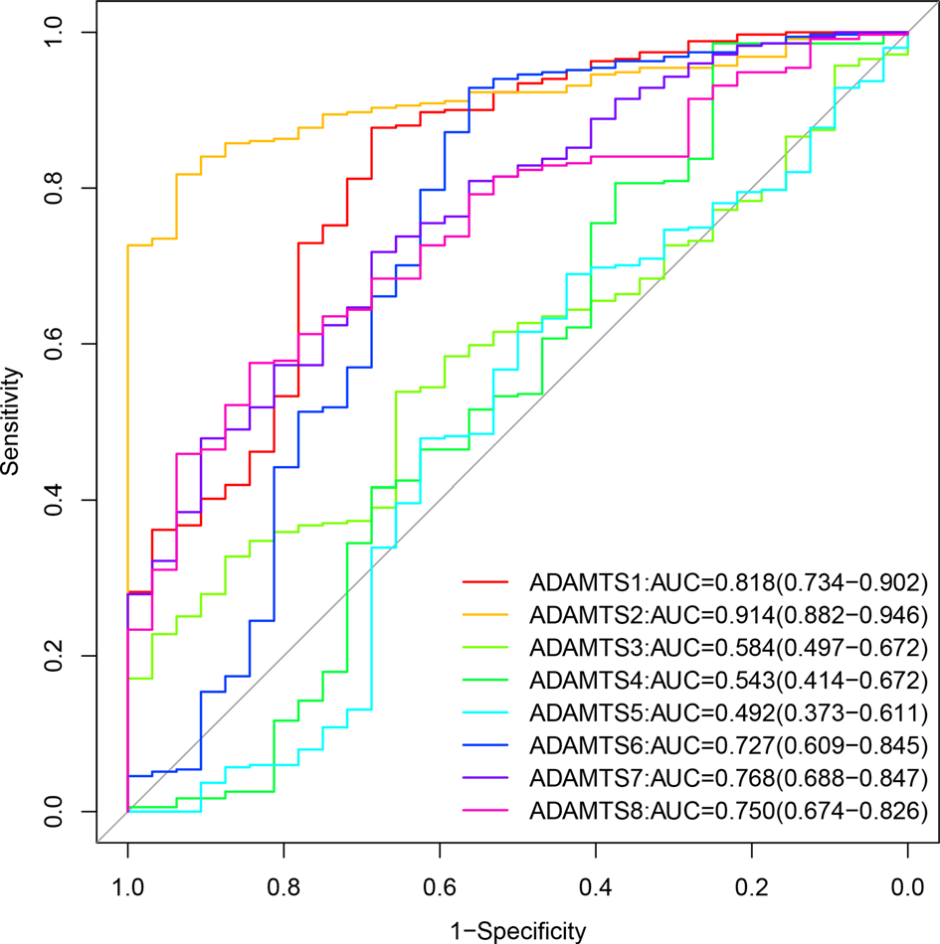
The ROC curve of *ADAMTS* mRNA expression in GC and adjacent tissues based on TCGA database

In determining the potential diagnostic value of ADAMTS family in distinguishing between GC tumor tissue and adjacent normal tissue, the results of unpaired *t* test showed that ADAMTS1 and ADAMTS8 mRNA expression was down-regulated in GC tumor tissues as compared with the adjacent normal tissues (*P*<0.05). On the other side, *ADAMTS*2, *ADAMTS*6, and *ADAMTS*7 genes mRNA expression were up-regulated in GC tissues (*P*<0.05), as shown in Figure 1. The ROC curves for the GC and adjacent normal samples showed that the tumor tissues could be effectively identified based on the risk score (Figure 2).

### Bioinformatics analysis

**Figure 3:**
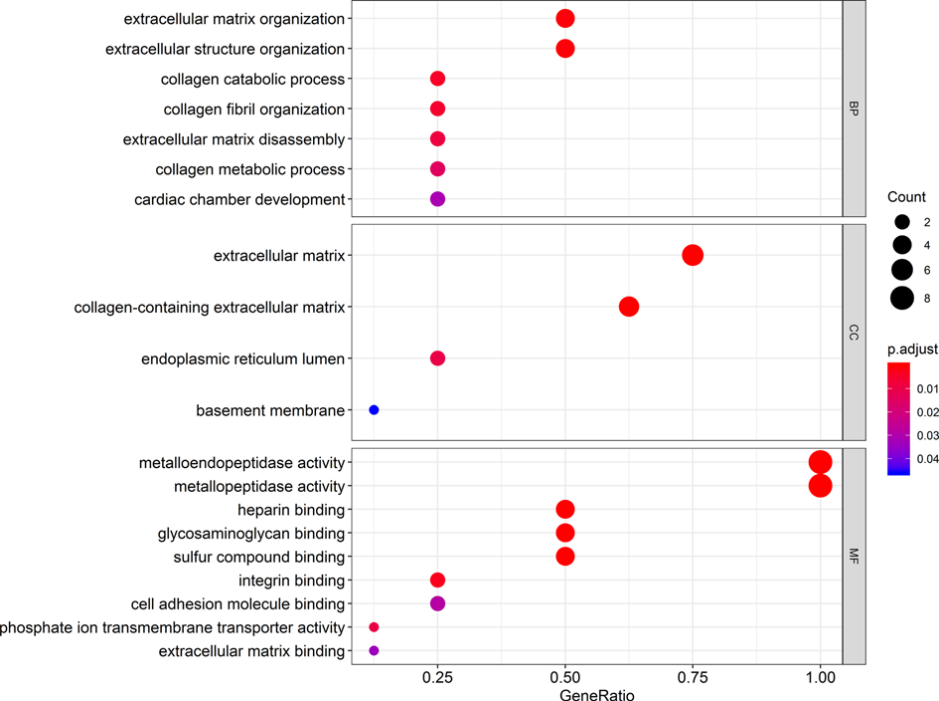
GO enrichment analysis of *ADAMTS* genes

**Figure 4:**
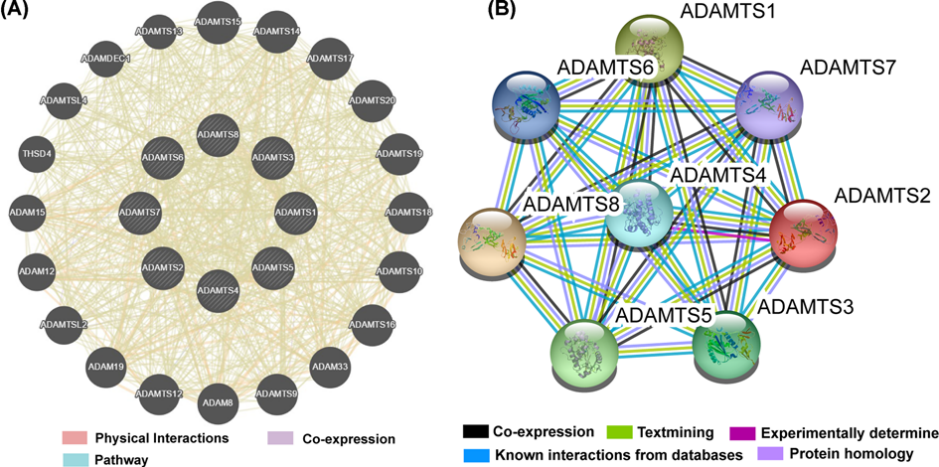
Gene and protein interaction networks of *ADAMTS* genes (**A**) Gene multiple association network integration algorithm. (**B**) PPI networks.

**Figure 5:**
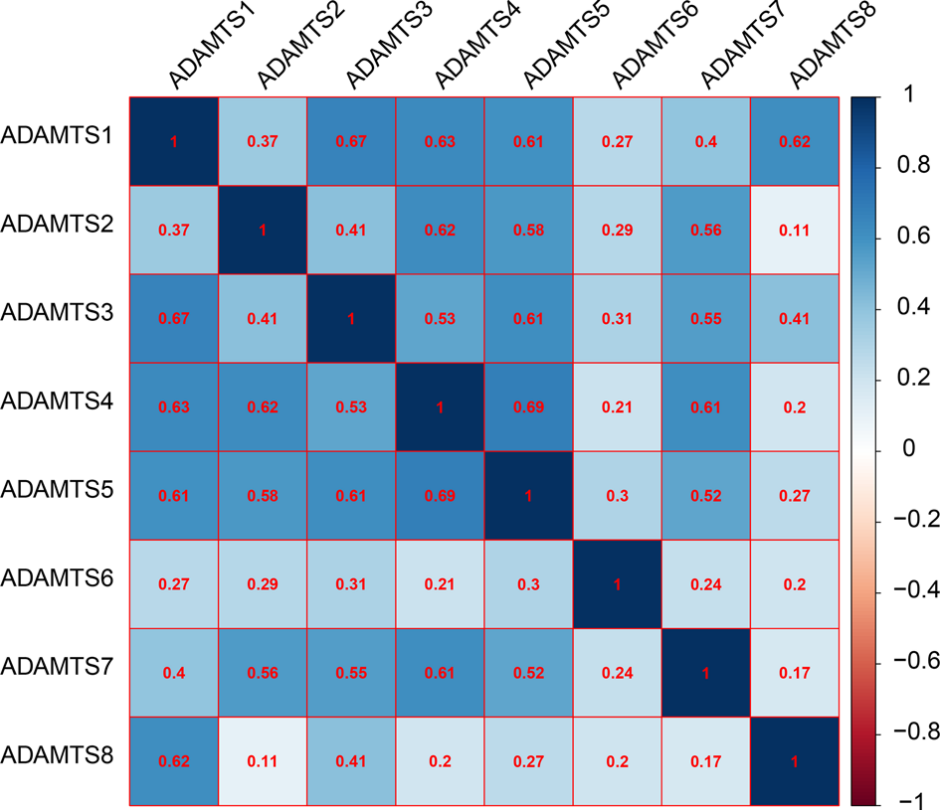
Co-expression matrix of *ADAMTS* genes in GC tumor tissues, and demonstrated that *ADAMTS*1–8 were positively correlated and co-expressed with each other in GC tumor tissues

**Figure 6:**
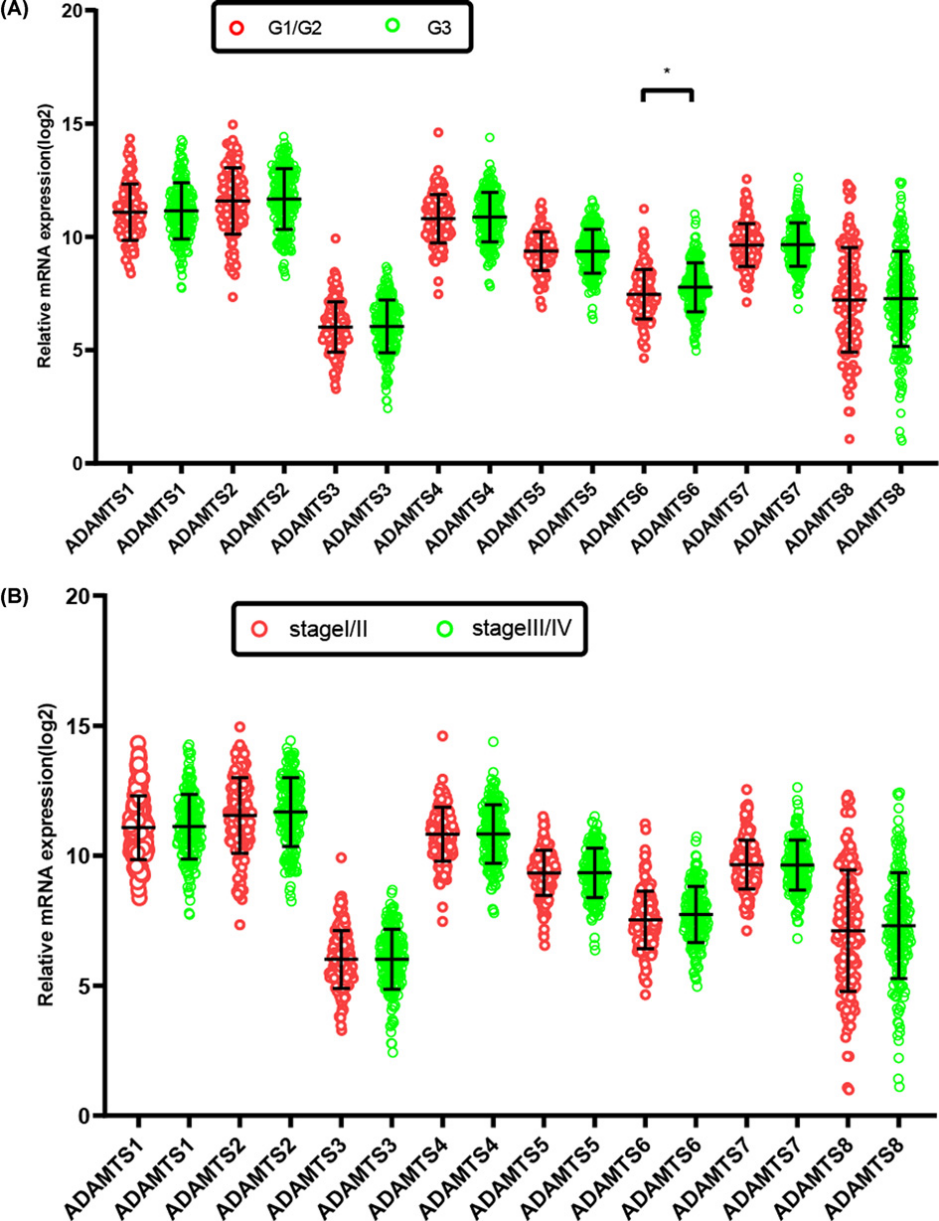
Gene expression distribution of *ADAMTS* genes in different GC histologic grades and tumor stages (**A**) Gene expression distribution of *ADAMTS* genes in different GC histologic grades. (**B**) Gene expression distribution of *ADAMTS* genes in different GC tumor stages. *P<0.05.

The GO analysis indicated that the *ADAMTS* genes were linked with extracellular matrix/structure organization, extracellular matrix disassembly, cell adhesion molecule binding, or collagen catabolic/metabolic processes, as shown in Figure 3. Our interaction network analysis showed that *ADAMTS* genes and other related genes formed an intricate network together. The gene–gene interaction network showed that the *ADAMTS* genes are strongly co-expressed (Figure 4A), while the PPI network analysis showed that the *ADAMTS* proteins directly interact with each other (Figure 4B). Furthermore, there was co-expression among the *ADAMTS*1–8 genes in the GC tumor tissues (Figure 5), demonstrating that the genes are positively correlated with GC development (Pearson correlation coefficient range: 0.11–0.69; *P*<0.05). Besides, our analysis of the *ADAMTS* genes expression in different tumor stages and histologic grades showed that the *ADAMTS*6 expression was significantly different in G1/G2 and G3 tumor tissues (Figure 6A). However, there was no significant difference in the expression of *ADAMTS* genes in stages I/II and III/IV tumor samples (Figure 6B). Together, these data show that *ADAMTS*6 overexpression is associated with G3 tumor tissues samples in GC patients, *P*=0.01 (Figure 6A).

### *ADAMTS*1–8 genes and OS in GC patients

**Figure 7:**
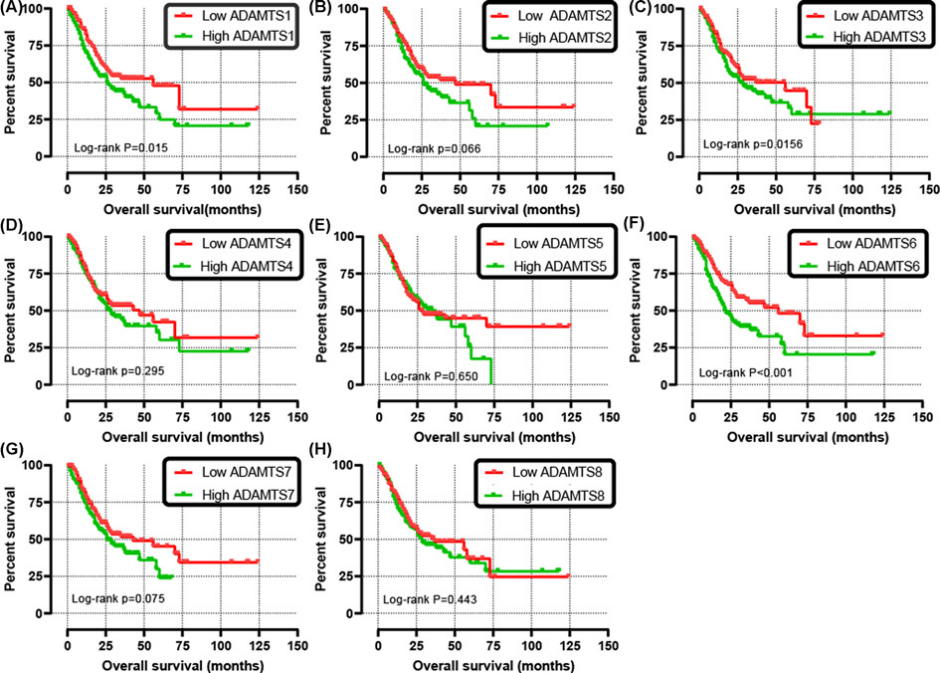
Kaplan–Meier survival curves for *ADAMTS* genes in GC of TCGA cohort OS stratified by *ADAMTS*1 (**A**), *ADAMTS*2 (**B**), *ADAMTS*3 (**C**), *ADAMTS*4 (**D**), *ADAMTS*5 (**E**), *ADAMTS*6 (**F**), *ADAMTS*7 (**G**), *ADAMTS*8 (**H**).

**Figure 8:**
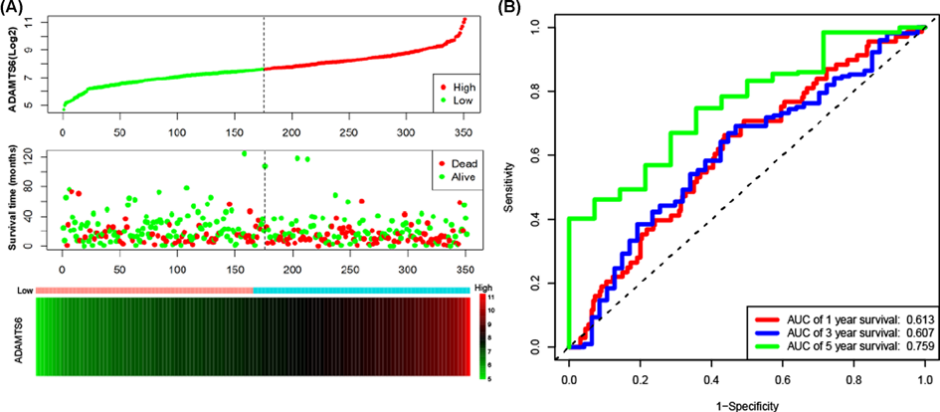
Prognostic value evaluation of *ADAMTS*6 in patients with GC (**A**) From top to bottom: are the expression values of *ADAMTS*6, patients’ survival status distribution, and the expression heat map of *ADAMTS*6 in the low- and high-expression groups. (**B**) ROC curve for predicting OS in GC patients by the *ADAMTS*6.

**Figure 9:**
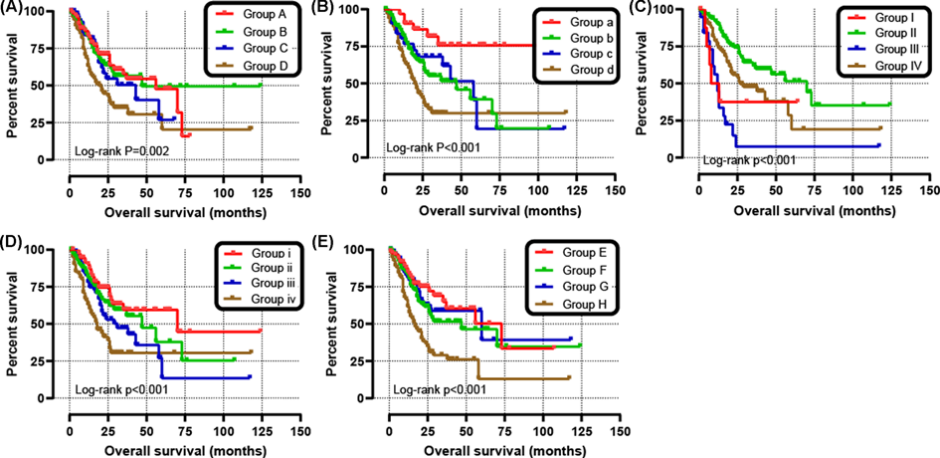
Joint effects analysis of OS stratified by *ADAMTS*6 and GC clinical parameters Joint effects analysis stratified by *ADAMTS*6 and following clinical parameters: histologic grade (**A**), radiation therapy (**B**), radical resection (**C**), targeted molecular therapy (**D**), stage (**E**).

The clinical characteristics of the 351 GC patients is shown in Table 1. The results from the univariate analysis found that the high *ADAMTS*1 and *ADAMTS*6 mRNA expression were associated with poor survival rates of GC patients (hazard ratio (HR) = 1.52, 95% confidence interval (CI) = 1.09–2.12, *P*=0.013 and HR = 1.80, 95% CI = 1.29–2.51, *P*=0.001, respectively) as shown in Table 2. Unlike the other *ADAMTS* genes, the survival analysis indicated that the up-regulation of *ADAMTS*1, *ADAMTS*3, or *ADAMTS*6 increased risk of death in GC patients (*P*<0.05) (Figure 7A–H). Clinical parameters such as age, TNM stage, cancer status, primary therapy outcome, residual tumor, and therapy were remarkably correlated with OS in GC. What needs to be adjusted in multivariate Cox regression and indicated that ADAMTS6 overexpression was remarkably raised death rate in GC (adjusted HR = 1.89, 95% CI = 1.19–3.01, adjusted *P*=0.007, Table 2, Figure 7F) and accelerated a worse OS (high *ADAMTS*6 vs low *ADAMTS*6; 21 vs 56 months, Table 2, Figures 7F and 8A). There was, however, no correlation between other *ADAMTS* genes and the GC OS. In addition, as shown in Table 3, subgroup analysis indicated that overexpression of the *ADAMTS*6 genes increased the death rate in GC patients who are ≥60 years old; female patients; patients with gastric antrum cancer; intestinal type adenocarcinoma; G2, G3 histologic grade; microsatellite instability-altitude (MSI-H), MMS; lymph node metastasis; T3, T4 stage; tumor free status; pathologic stage III; R0 resection; those untreated with radiation therapy or targeted therapy, without distant metastasis and HP infection. Time-dependent ROC analysis proved that *ADAMTS*6 expression profile could reliably predict OS in GC patients. The area under the 1-, 2-, or 3-year curve (AUC) was 0.613, 0.607, or 0.759, respectively (Figure 8B). Besides, we demonstrate that *ADAMTS*6, together with the OS-related clinical characteristics give superior performance in the prediction OS in GC patients (Figure 9A–E and Table 4).

**Table 1:** Correlation between OS and clinicopathologic features of GC patients in TCGA cohort

Variables	Events/total	MST (months)	HR (95% CI)	*P*-value
Age (years)	144/348	29		0.022
<60	36/108	60	1	
≥60	108/240	26	1.55 (1.06–2.27)	
Missing	3			
Gender	144/351	29		0.178
Male	100/226	29	1	
Female	44/125	35	0.78 (0.55–1.12)	
Missing	0			
Anatomic neoplasm	138/337	29		0.919
Gastroesophageal junction	36/84	26	1	
Fundus gastric body	50/123	28	0.92 (0.60–1.41)	
Gastric antrum	52/130	35	0.94 (0.61–1.43)	
Missing	14			
HP infection	66/161	43		0.304
Positive	6/18	58	1	
Negative	60/143	43	1.55 (0.67–3.61)	
Missing	190			
Histologic type	144/350	29		0.057
Intestinal	64/160	38	1	
Diffuse type	24/61	60	1.00 (0.63–1.60)	
Signet ring type	8/11	13	2.52 (1.20–5.25)	
Other types	48/118	26	1.29 (0.88–1.88)	
Missing	1			
Histologic grade	140/342	29		0.169
G1	2/9	NA	1	
G2	48/127	43	1.67 (0.41–6.86)	
G3	90/206	26	2.22 (0.55–9.01)	
Missing	9			
MSS1	144/350	29		0.225
MSI-H	99/240	28	1	
MSI-L	22/51	29	1.26 (0.79–2.01)	
MMS	23/59	35	0.76 (0.48–1.19)	
Missing	1			
Pathological M	138/336	29		0.012
M1	13/23	12	1	
M0	125/313	35	0.49 (0.28–0.86)	
Missing	15			
Pathological N	139/341	29		0.004
N0	28/103	60	1	
N+	111/238	25	1.83 (1.21–2.76)	
Missing	10			
Pathological T	140/347	31		0.009
T1/T2	28/91	70	1	
T3/T4	112/256	26	1.73 (1.14–2.63)	
Missing	4			
TNM stage	136/338	29		<0.001
Stage I	11/47	73	1	
Stage II	34/109	56	1.61 (0.81–3.18)	
Stage III	69/147	26	2.44 (1.29–4.61)	
Stage IV	22/35	16	3.79 (1.84–7.82)	
Missing	13			
Cancer status	121/324	37		<0.001
Tumor free	35/206	NA	1	
With tumor	86/118	17	5.53 (3.70–8.26)	
Missing	27			
Primary therapy outcome	114/303	38		<0.001
CR	55/209	73	1	
PR	4/5	17	4.23 (1.52–11.78)	
SD	7/25	31	1.89 (0.86–4.18)	
PD	48/64	13	4.33 (2.91–6.45)	
Missing	47			
Radiation therapy	135/328	31		0.001
Yes	19/62	NA	1	
No	116/266	26	2.32 (1.42–3.80)	
Missing	23			
Residual tumor	121/316	37		<0.001
R1	9/14	13	1	
R2	12/14	9	1.88 (0.95–3.73)	
R0	100/288	47	7.19 (3.88–13.34)	
Missing	35			
Targeted therapy	134/326	31		0.022
Yes	56/151	43	1	
No	78/175	26	1.49 (1.06–2.10)	
Missing	25			

Abbreviations: CR, complete response; G1, highly differentiated; G2, moderately differentiated; G3, poorly differentiated; MSI-L, microsatellite instability-altitude; MST, median survival time; PD, progressive disease; PR, partial response; R0, no residual tumor; R1, microscopic residual tumor; R2, macroscopic residual tumor; SD, stable disease.

**Table 2:** Prognostic values of *ADAMTS* genes expression in GC OS of TCGA cohort

Gene expression	Events/total (*n*=351)	MST (months)	Crude HR (95% CI)	Crude *P*-value	Adjusted HR (95% CI)	Adjusted *P*-value^1^
*ADAMTS*1						
Low	61/176	56	1		1	
High	83/175	26	1.52 (1.09–2.12)	0.013	1.39 (0.91–2.11)	0.125
*ADAMTS*2						
Low	64/176	47	1		1	
High	80/175	26	1.38 (0.99–1.92)	0.055	1.30 (0.85–1.98)	0.226
*ADAMTS*3						
Low	63/176	37	1		1	
High	81/175	27	1.25 (0.90–1.73)	0.19	1.36 (0.88–2.09)	0.168
*ADAMTS*4						
Low	63/176	43	1		1	
High	81/175	28	1.21 (0.87–1.68)	0.266	1.34 (0.87–2.07)	0.189
*ADAMTS*5						
Low	70/176	27	1		1	
High	74/175	35	1.09 (0.79–1.52)	0.599	0.84 (0.54–1.31)	0.441
*ADAMTS*6						
Low	59/176	56	1		1	
High	85/175	21	1.80 (1.29–2.51)	0.001	1.89 (1.19–3.01)	0.007
*ADAMTS*7						
Low	64/176	43	1		1	
High	80/175	26	1.33 (0.95–1.85)	0.095	1.41 (0.91–2.18)	0.120
*ADAMTS*8						
Low	67/176	37	1		1	
High	77/175	28	1.15 (0.83–1.60)	0.401	1.52 (0.97–2.39)	0.067

Abbreviation: MST, median survival time.

1 Adjusted for age, TNM stage, cancer status, primary therapy outcome, residual tumor, targeted molecular therapy, and radiation therapy.

**Table 3:** Stratified analysis of *ADAMTS*6 gene expression in clinicopathologic features of GC cases

Variables	Cases	HR (95% CI)	Log-rank *P*-value
Age (years)	348		
<60	108	1.01 (0.46–2.21)	0.102
≥60	240	1.94 (1.25–3.01)	0.048
Gender	351		
Male	226	1.66 (1.05–2.62)	0.021
Female	125	2.26 (1.14–4.50)	0.293
Anatomic neoplasm			
Gastroesophageal	337		
Junction	84	0.79 (0.29–2.15)	0.501
Fundus gastric body	123	0.70 (0.39–1.24)	0.001
Gastric antrum	130	1.97 (1.01–3.85)	0.001
HP infection	161		
Positive	18	3.61 (0.22–59.81)	0.180
Negative	143	1.20 (0.65–2.23)	0.005
Histologic type	350		
Intestinal	160	2.53 (1.43–4.46)	*P*<0.001
Diffuse type	61	0.67 (0.28–1.59)	0.158
Signet ring type	11	0.64 (0.02–26.22)	0.312
Other types	118	1.96 (1.01–3.82)	0.005
Histologic grade	342		
G1	9	NA	NA
G2	127	1.15 (0.59–2.26)	0.006
G3	206	2.38 (1.49–3.81)	*P*<0.001
MSS1	350		
MSI-H	240	1.67 (1.08–2.59)	*P*<0.001
MSI-L	51	2.15 (0.81–5.73)	0.099
MMS	59	2.62 (0.88–7.81)	0.005
Pathological M	336		
M1	23	1.19 (0.07–19.35)	0.870
M0	313	1.63 (1.12–2.38)	*P*<0.001
Pathological N	139/341		
N0	103	0.90 (0.35–2.30)	0.005
N+	111/238	2.20 (1.41–3.42)	*P*<0.001
Pathological T	347		
T1/T2	91	0.60 (0.24–1.51)	0.118
T3/T4	256	2.24 (1.44–3.50)	*P*<0.001
Pathologic stage	136/338		
Stage I	47	1.60 (0.48–5.29)	0.402
Stage II	109	1.55 (0.79–3.07)	0.478
Stage III	147	2.00 (1.16–3.44)	0.039
Stage IV	35	3.11 (0.70–13.86)	0.132
Cancer status	121/324		
Tumor free	206	2.26 (1.07–4.80)	*P*<0.001
With tumor	118	1.71 (1.06–2.76)	0.108
Radiation therapy	135/328		
Yes	62	2.26 (0.80–6.39)	0.287
No	266	1.75 (1.78–2.59)	*P*<0.001
Residual tumor	121/316		
R1	14	NA	NA
R2	14	0.78 (0.02–41.15)	0.531
R0	288	1.71 (1.13–2.58)	0.090
Targeted therapy	134/326		
Yes	151	1.36 (0.77–2.39)	0.388
No	175	1.92 (1.18–3.12)	*P*<0.001

Abbreviations: CR, complete response; G1, highly differentiated; G2, moderately differentiated; G3, poorly differentiated; MSI-L, microsatellite instability-low; MST, median survival time; M, metastasis; N, node; NA, not applicable; PD, progressive disease; PR, partial response; R0, no residual tumor; R1, microscopic residual tumor; R2, macroscopic residual tumor; SD, stable disease; T, tumor.

**Table 4:** Joint effects survival analysis of clinical factors and the *ADAMTS*6 expression with OS

Group	*ADAMTS*6	Variables	Events/total	MST (months)	Crude HR (95% CI)	Crude *P*-value	Adjusted HR (95% CI)	Adjusted *P*-value^1^
Histologic grade								
A	Low expression	G1 + G2	27/74	56	1		1	
B	Low expression	G3 + G4	32/98	47	1.005 (0.602–1.680)	0.984	0.734 (0.394–1.371)	0.332
C	High expression	G1 + G2	23/62	31	I.226 (0.701–2.142)	0.475	1.171 (0.600–2.286)	0.644
D	High expression	G3 + G4	58/108	18	2.045 (1.291–3.237)	0.002	1.815 (1.035–3.184)	0.038
Radiation therapy								
a	Low expression	Yes	6/31	47	1		1	
b	Low expression	No	47/131	58	3.104 (1.321–7.2940	0.009	3.265 (1.276–8.353)	0.014
c	High expression	Yes	13/31	20	2.821 (1.071–7.432)	0.036	2.644 (0.954–7.324)	0.062
d	High expression	No	69/135	31	5.665 (2.434–13.188)	<0.001	5.917 (2.349–14.900)	<0.001
Radical resection								
I	Low expression	R0	44/155	70	1		1	
II	Low expression	R1+R2	5/9	8	3.046 (1.204–7.707)	0.019	3.308 (0.931–11.751)	0.064
IV	High expression	R0	56/133	27	1.829 (1.230–2.720)	0.003	2.055 (1.349–3.129)	0.001
III	High expression	R1+R2	16/19	12	5.009 (2.807–8.937)	<0.001	3.485 (1.719–7.067)	0.001
Targeted molecular therapy								
i	Low expression	Yes	22/74	70	1		1	
ii	Low expression	No	31/89	47	1.400 (0.810–2.420)	0.228	0.928 (0.478–1.801)	0.825
iii	High expression	Yes	34/77	29	1.829 (1.068–3.133)	0.028	1.626 (0.891–2.966)	0.113
iv	High expression	No	47/86	18	3.002 (1.802–5.001)	<0.001	2.030 (1.087–3.793)	0.026
Stage								
E	Low expression	I + II	24/89	56	1		1	
F	Low expression	III+IV	34/84	47	1.398 (0.829–2.358)	0.209	1.839 (0.975–3.469)	0.06
G	High expression	I + II	21/67	60	1.214 (0.675–2.181)	0.517	1.579 (0.794–3.142)	0.193
H	High expression	III+IV	57/98	17	2.916 (1.807–4.704)	<0.001	3.897 (2.117–7.176)	<0.001

Abbreviation: MST, median survival time.

1 Adjusted for histologic grade, radiation therapy, radical resection, targeted molecular therapy, and stage.

### K–M plotter survival analysis

**Figure 10:**
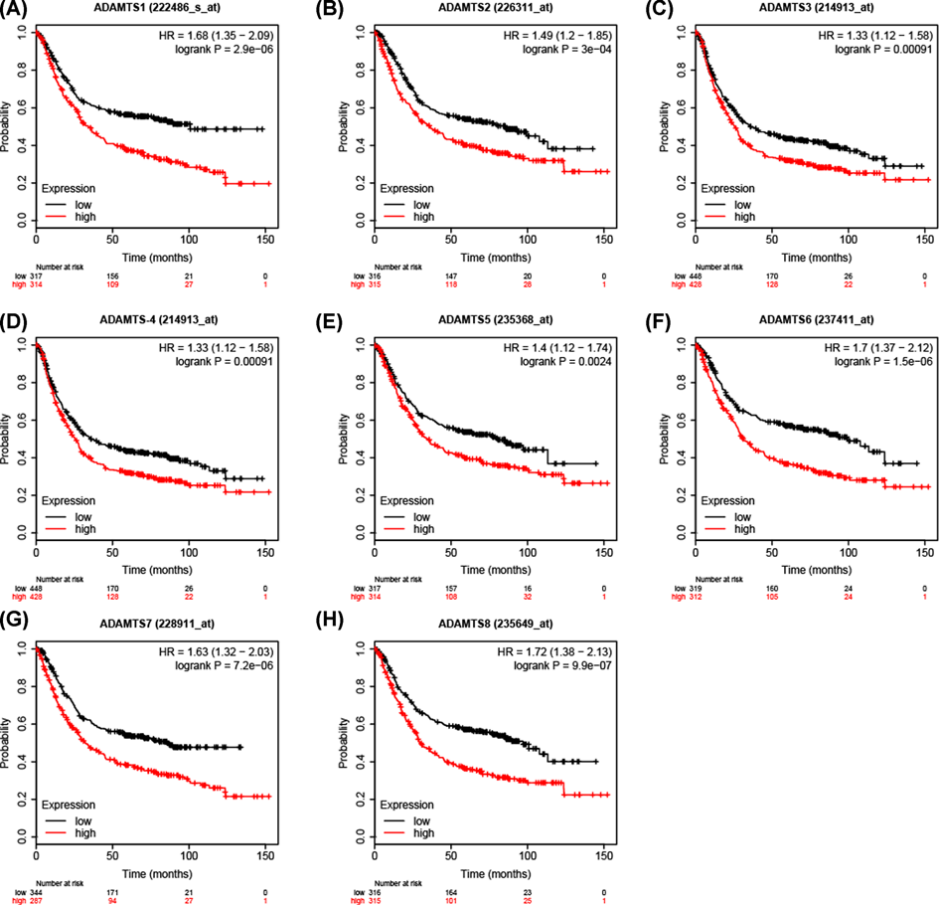
Survival analysis of *ADAMTS* mRNA expression in GC based on K–M plotter database Survival analysis of *ADAMTS*1 (**A**), *ADAMTS*2 (**B**), *ADAMTS*3 (**C**), *ADAMTS*4 (**D**), *ADAMTS*5 (**E**), *ADAMTS*6 (**F**), *ADAMTS*7 (**G**), *ADAMTS*8 (**H**).

**Figure 11:**
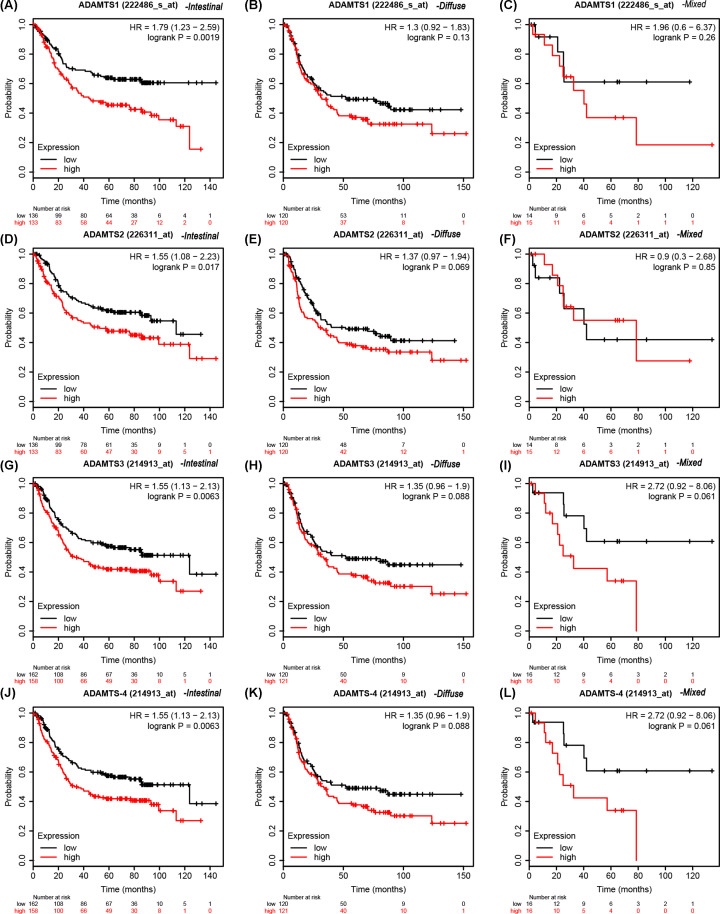
Stratified analysis of *ADAMTS*1–4 gene mRNA in Lauren typing of GC cases in K–M plotter database OS curves for (**A**) intestinal-type ADAMTS1, (**B**) diffuse-type ADAMTS1, (**C**) mixed-type ADAMTS1, (**D**) intestinal-type ADAMTS2, (**E**) diffuse-type ADAMTS2, (**F**) mixed-type ADAMTS2, (**G**) intestinal-type ADAMTS3, (**H**) diffuse-type ADAMTS3, (**I**) mixed-type ADAMTS3, (**J**) intestinal-type ADAMTS4, (**K**) diffuse-type ADAMTS4, (**L**) mixed-type ADAMTS4.

**Figure 12:**
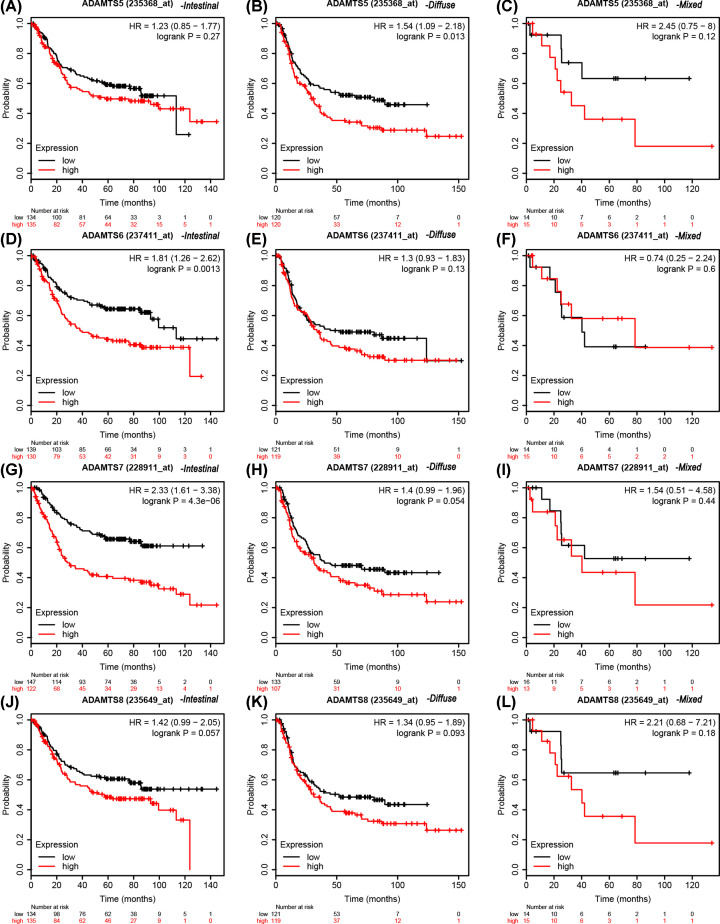
Stratified analysis of *ADAMTS*5-8 gene mRNA in Lauren typing of GC cases in K–M plotter database OS curves for (**A**) intestinal-type ADAMTS5, (**B**) diffuse-type ADAMTS5, (**C**) mixed-type ADAMTS5, (**D**) intestinal-type ADAMTS6, (**E**) diffuse-type ADAMTS6, (**F**) mixed-type ADAMTS6, (**G**) intestinal-type ADAMTS7, (**H**) diffuse-type ADAMTS7, (**I**) mixed-type ADAMTS7, (**J**) intestinal-type ADAMTS8, (**K**) diffuse-type ADAMTS8, (**L**) mixed-type ADAMTS8.

The expression profile for the *ADAMTS* genes in the K–M plotter database demonstrated that patients with high expression had poor clinical outcomes (Figure 10 and Table 5). The data from the Lauren classification of the *ADAMTS* mRNA expression of the GC patients showed that the patients with high expression of diffuse *ADAMTS*5 (Affymetrix ID: 235368_at) gene, intestinal *ADAMTS*1 (Affymetrix ID: 222486_s_at), *ADAMTS*2 (Affymetrix ID: 226311_at), *ADAMTS*3 (Affymetrix ID: 214913_at), *ADAMTS*4 (Affymetrix ID: 214913_at), *ADAMTS*6 (Affymetrix ID: 237411_at) or *ADAMTS*7 (Affymetrix ID: 228911_at) had shorter survival time compared with those with low expression (Figures 11 and 12 and Table 6). Tables 7-10 show the stratified results of the *ADAMTS* expression in TNM stage, tumor differentiation degree, different treatment strategies, and HER2 status of the GC cases. The high expression of *ADAMTS*1 in tumor stage IV (adjusted *P*=0.036, adjusted HR = 1.52, 95% CI = 1.02−2.27), *ADAMTS*2 in stage III (adjusted *P*=0.02, adjusted HR = 1.56, 95% CI = 1.07−2.28), *ADAMTS*5 in stages III and IV (adjusted *P*=0.002, adjusted HR = 1.81, 95% CI = 1.24−2.64 and adjusted *P*=0.04, adjusted HR = 1.51, 95% CI = 1.02−2.25), *ADAMTS*7 in stages III and IV (adjusted *P*=0.0026, adjusted HR = 1.77, 95% CI = 1.21−2.57 and adjusted *P*=0.0047, adjusted HR = 1.76, 95% CI = 1.18−2.63) increased the mortality rate, while a high expression of *ADAMTS*2 in tumor stage I (adjusted *P*=0.03, adjusted HR = 0.26, 95% CI = 0.07−0.96) increased the survival rate in GC patients. Similarly, the high expression in G2 degree of tumor differentiation of *ADAMTS*7 (adjusted *P*=0.0074, adjusted HR = 2.39, 95% CI = 1.24−4.59) increased the death rate in GC patients. In addition, the mortality rate in patients with high expression of *ADAMTS* genes after surgery was higher than those with low expression. The HER2 state subgroup analysis showed that the high expression of *ADAMTS* family of genes increased mortality in patients with HER2 positive and HER2 negative status, but not in *ADAMTS*5, *ADAMTS*7 HER2 positive patients.

**Table 5:** Survival analysis of *ADAMTS* gene mRNA expression in GC cases in KM plotter database

Gene/Affymetrix ID	Low/high expression cases	HR (95% CI)	*P*-value
*ADAMTS*1/222486_s_at	317/314	1.68 (1.35–2.09)	2.9e-06
*ADAMTS*2/226311_at	316/315	1.49 (1.2–1.85)	3e-04
*ADAMTS*3/214913_at	448/428	1.33 (1.12–1.58)	0.00091
*ADAMTS*4/214913_at	448/428	1.33 (1.12–1.58)	0.00091
*ADAMTS*5/235368_at	317/314	1.4 (1.12–1.74)	0.0024
*ADAMTS*6/237411_at	319/312	1.7 (1.37–2.12)	1.5e-06
*ADAMTS*7/228911_at	344/287	1.63 (1.32–2.03)	7.2e-06
*ADAMTS*8/235649_at	316/315	1.72 (1.38–2.13)	9.9e-07

**Table 6:** Stratified analysis of *ADAMTS* gene mRNA in Lauren typing in K–M plotter

Gene	Lauren typing	Low/high expression cases	HR (95% CI)	*P*-value
*ADAMTS*1	Intestinal	136/133	1.79 (1.23–2.59)	0.0019
	Diffuse	120/120	1.3 (0.92–1.83)	0.13
	Mixed	14/15	1.96 (0.6–6.37)	0.26
*ADAMTS*2	Intestinal	136/133	1.55 (1.08–2.23)	0.017
	Diffuse	120/120	1.37 (0.97–1.94)	0.069
	Mixed	14/15	0.9 (0.3–2.68)	0.85
*ADAMTS*3	Intestinal	162/158	1.55 (1.13–2.13)	0.0063
	Diffuse	120/121	1.35 (0.96–1.9)	0.088
	Mixed	16/16	2.72 (0.92–8.06)	0.061
*ADAMTS*4	Intestinal	162/158	1.55 (1.13–2.13)	0.0063
	Diffuse	120/121	1.35 (0.96–1.9)	0.088
	Mixed	16/16	2.72 (0.92–8.06)	0.061
*ADAMTS*5	Intestinal	134/135	1.23 (0.85–1.77)	0.27
	Diffuse	120/120	1.54 (1.09–2.18)	0.013
	Mixed	14/15	0.9 (0.3–2.68)	0.85
*ADAMTS*6	Intestinal	139/130	1.81 (1.26–2.62)	0.0013
	Diffuse	121/119	1.3 (0.93–1.83)	0.13
	Mixed	14/15	0.74 (0.25–2.24)	0.6
*ADAMTS*7	Intestinal	147/122	2.33 (1.61–3.38)	4.3e-06
	Diffuse	133/107	1.4 (0.99–1.96)	0.054
	Mixed	16/13	1.54 (0.51–4.58)	0.44
*ADAMTS*8	Intestinal	134/135	1.42 (0.99–2.05)	0.057
	Diffuse	121/119	1.34 (0.95–1.89)	0.093
	Mixed	14/15	2.21 (0.68–7.21)	0.18

**Table 7:** Stratified analysis of *ADAMTS* gene mRNA in stage in K–M plotter database

Gene	Stage	Low/high expression cases	HR (95% CI)	*P*-value
*ADAMTS*1	I	31/31	1.95 (0.6–6.4)	0.26
	II	69/66	1.57 (0.83–2.97)	0.16
	III	98/99	1.11 (0.77–1.62)	0.57
	IV	70/70	1.52 (1.02–2.27)	0.036
*ADAMTS*2	I	31/31	0.26 (0.07–0.96)	0.03
	II	68/67	0.62 (0.33–1.19)	0.15
	III	98/99	1.56 (1.07–2.28)	0.02
	IV	70/70	1.29 (0.87–1.91)	0.2
*ADAMTS*3	I	35/32	1.18 (0.43–3.23)	0.75
	II	70/70	1.52 (0.83–2.76)	0.17
	III	152/153	1.13 (0.85–1.5)	0.4
	IV	74/74	1.46 (1–2.15)	0.051
*ADAMTS*4	I	35/32	1.18 (0.43–3.23)	0.75
	II	70/70	1.52 (0.83–2.76)	0.17
	III	152/153	1.13 (0.85–1.5)	0.4
	IV	74/74	1.46 (1–2.15)	0.051
*ADAMTS*5	I	31/31	0.48 (0.16–1.49)	0.19
	II	68/67	1.66 (0.86–3.18)	0.13
	III	98/99	1.81 (1.24–2.64)	0.002
	IV	71/69	1.51 (1.02–2.25)	0.04
*ADAMTS*6	I	32/30	0.41 (0.12–1.33)	0.12
	II	68/67	1.4 (0.74–2.63)	0.29
	III	98/99	1.36 (0.93–1.98)	0.11
	IV	70/70	1.31 (0.88–1.95)	0.18
*ADAMTS*7	I	34/28	2.56 (0.77–8.56)	0.11
	II	82/53	1.35 (0.71–2.57)	0.35
	III	110/87	1.77 (1.21–2.57)	0.0026
	IV	74/66	1.76 (1.18–2.63)	0.0047
*ADAMTS*8	I	31/31	1.87 (0.56–6.23)	0.3
	II	68/67	1.52 (0.8–2.88)	0.19
	III	98/99	1.2 (0.82–1.74)	0.34
	IV	70/70	1.4 (0.94–2.09)	0.096

**Table 8:** Stratified analysis of *ADAMTS* gene mRNA in degree of tumor differentiation in K–M plotter

Gene	Differentiation	Low/high expression cases	HR (95% CI)	*P*-value
*ADAMTS*1	G1	2/3	1142066032.99 (0–Inf)	0.41
	G2	34/33	0.89 (0.46–1.7)	0.72
	G3	60/61	0.87 (0.53–1.4)	0.55
*ADAMTS*2	G1	2/3	1142066039.57 (0–Inf)	0.41
	G2	34/33	1.31 (0.69–2.52)	0.41
	G3	60/61	1.34 (0.82–2.18)	0.24
*ADAMTS*3	G1	16/16	1.52 (0.64–3.61)	0.34
	G2	34/33	1.35 (0.71–2.59)	0.36
	G3	82/83	1.23 (0.82–1.83)	0.32
*ADAMTS*4	G1	16/16	1.52 (0.64–3.61)	0.34
	G2	34/33	1.35 (0.71–2.59)	0.36
	G3	82/83	1.23 (0.82–1.83)	0.32
*ADAMTS*5	G1	2/3	1142066032.99 (0–Inf)	0.41
	G2	34/33	0.96 (0.5–1.84)	0.91
	G3	60/61	1.16 (0.72–1.88)	0.54
*ADAMTS*6	G1	2/3	1142066039.57 (0–Inf)	0.41
	G2	36/31	1.49 (0.78–2.84)	0.23
	G3	62/59	1.07 (0.66–1.73)	0.78
*ADAMTS*7	G1	2/3	1142066042.6 (0–Inf)	0.41
	G2	40/27	2.39 (1.24–4.59)	0.0074
	G3	60/61	0.77 (0.48–1.26)	0.3
*ADAMTS*8	G1	2/3	1142066039.57 (0–Inf)	0.41
	G2	34/33	1.43 (0.75–2.74)	0.28
	G3	60/61	1.1 (0.68–1.79)	0.69

**Table 9:** Stratified analysis of *ADAMTS* genes mRNA in treatment method in K–M plotter

Gene	Treatment method	Low/high expression cases	HR (95% CI)	*P*-value
*ADAMTS1*	Surgery	189/191	1.66 (1.24–2.22)	0.00065
	5-FU	17/17	0.99 (0.4–2.47)	0.98
*ADAMTS*2	Surgery	192/188	1.32 (0.99–1.76)	0.061
	5-FU	17/17	2.26 (0.89–5.76)	0.08
*ADAMTS*3	Surgery	194/186	1.54 (1.15–2.06)	0.0032
	5-FU	77/76	0.88 (0.63–1.24)	0.47
*ADAMTS*4	Surgery	194/186	1.54 (1.15–2.06)	0.0032
	5-FU	77/76	0.88 (0.63–1.24)	0.47
*ADAMTS*5	Surgery	190/190	1.28 (0.96–1.71)	0.096
	5-FU	17/17	0.68 (0.27–1.71)	0.42
*ADAMTS*6	Surgery	190/190	1.75 (1.31–2.34)	0.00014
	5-FU	17/17	2.04 (0.81–5.16)	0.12
*ADAMTS*7	Surgery	209/171	1.57 (1.17–2.09)	0.0021
	5-FU	17/17	1 (0.4–2.47)	1
*ADAMTS*8	Surgery	190/190	1.48 (1.11–1.98)	0.0075
	5-FU	17/17	2.25 (0.85–5.95)	0.094

**Table 10:** Stratified analysis of *ADAMTS* gene mRNA in HER2 state in K–M plotter database

Gene	HER2 state	Low/high expression cases	HR (95% CI)	*P*-value
*ADAMTS*1	Negative	214/215	1.6 (1.23–2.1)	0.00049
	Positive	101/101	1.94 (1.32–2.84)	0.00063
*ADAMTS*2	Negative	214/215	1.46 (1.12–1.91)	0.0049
	Positive	101/101	1.59 (1.09–2.32)	0.015
*ADAMTS*3	Negative	266/266	1.49 (1.19–1.87)	0.00053
	Positive	172/172	1.26 (0.97–1.63)	0.082
*ADAMTS*4	Negative	266/266	1.49 (1.19–1.87)	0.00053
	Positive	172/172	1.26 (0.97–1.63)	0.082
*ADAMTS*5	Negative	216/213	1.44 (1.1–1.87)	0.0077
	Positive	102/100	1.37 (0.94–1.99)	0.1
*ADAMTS*6	Negative	215/214	1.75 (1.33–2.29)	4.2e-05
	Positive	101/101	1.59 (1.09–2.31)	0.015
*ADAMTS*7	Negative	225/204	1.85 (1.42–2.43)	4.6e-06
	Positive	104/98	1.33 (0.92–1.93)	0.13
*ADAMTS*8	Negative	214/215	1.75 (1.34–2.29)	3.7e-05
	Positive	101/101	1.64 (1.12–2.38)	0.0096

### GSEA

**Figure 13:**
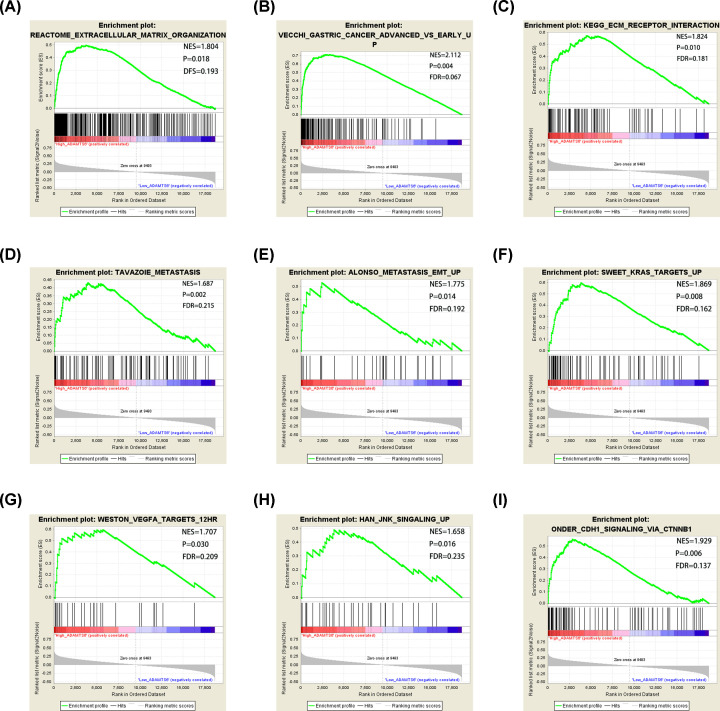
GSEA of C2 gene sets for high *ADAMTS*6 expression groups (**A**) ‘Extracellular matrix organization’, (**B**) ‘Gastric cancer advanced vs early up’, (**C**) ‘ECM receptor interaction’, (**D**) ‘Tavazoie metastasis’, (**E**) ’Alonso metastasis EMT up’, (**F**) ‘KRAS targets up’ (**G**) ‘VEGFA targets 12HR’, (**H**) ‘JNK signaling up’ and (**I**) ‘CDH1 signaling pathway’ were enriched in the ADAMTS6 high-expression groups.

**Figure 14:**
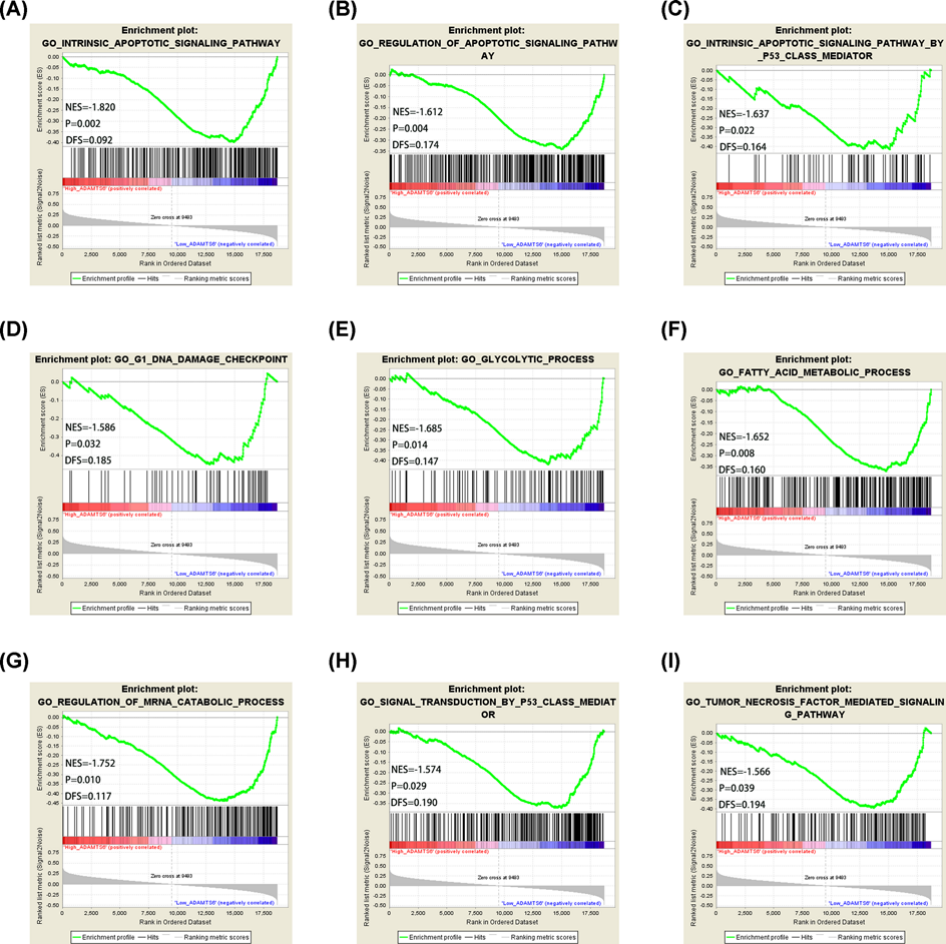
GSEA of C5 gene sets for low *ADAMTS*6 expression groups The GO terms (**A**) ‘Intrinsic apoptotic signaling pathway’, (**B**) ‘Regulation of apoptotic signaling pathway’, (**C**) ‘Intrinsic apoptotic signaling pathway by P53 class mediator’, (**D**) ‘G1 DNA damage checkpoint’, (**E**) ‘Glycolysis process’, (**F**) ‘Fatty acid metabolic process’, (**G**) ‘Regulation of mRNA catabolic process’, (**H**) ‘Signal transduction by P53 class mediator’ and (**I**) ‘Tumor necrosis factor-mediated signaling pathways’ were enriched in the ADAMTS6 low-expression groups.

The GSEA data indicated that, in the c2 category, the high expression of *ADAMTS*6 may participate in extracellular matrix organization, development of advanced GC, metastasis, ECM receptor interaction, vascular endothelial growth factor A (VEGFA), kirsten rat sarcoma viral oncogene (KRAS), c-Jun N-terminal kinase (JNK), and cadherin (CDH1) signaling pathways (Figure 13A–I). In the c5 category, down-regulation of the *ADAMTS*6 expression might be linked with DNA damage, cell cycle, apoptosis, glycolysis, fatty acid metabolism, mRNA catabolism, tumor protein p53, and tumor necrosis factor (TNF) signaling pathways (Figure 14A–I).

### qRT-PCR and immunohistochemistry

**Figure 15:**
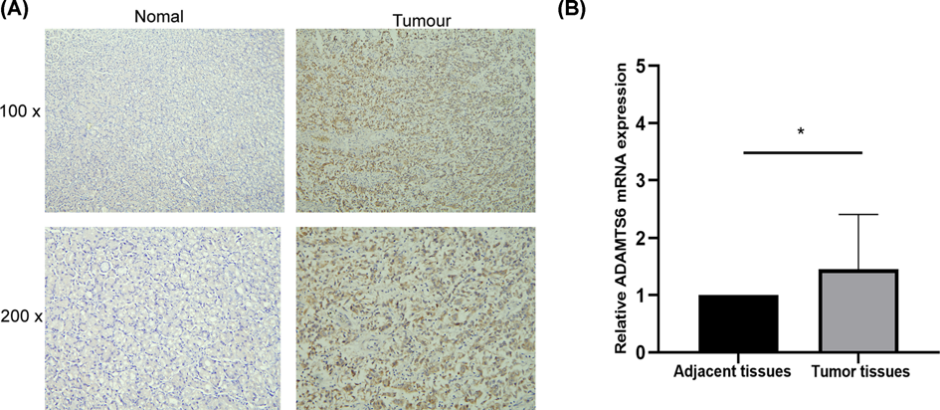
ADAMTS6 expression in GC patients (**A**) Representative images of immunostaining for ADAMTS6 in GC and adjacent normal tissues. (**B**) ADAMTS6 levels were detected in 24 pairs of GC tissues by qRT-PCR, revealing higher ADAMTS6 expression in GC tissues relative to paracancerous tissues. *P<0.05.

Although the application of bioinformatics has given us directions, we still needed to combine sample verification to provide guidance for clinical research. We found that ADAMTS6 is mainly expressed in the cytoplasm of GC tissues. The positive expression of ADAMTS6 was observed in 10 (55.56%) and 4 (22.22%) cases of GC and adjacent normal tissues, respectively (Table 11, *P*<0.05), which proved that ADAMTS6 was up-regulated in GC compared with adjacent tissues (Figure 15A,B), consistent with TCGA findings (Figure 1, *P*<0.05).

**Table 11:** ADAMTS6 expression in GC and paracarcioma tissues

	ADAMTS6 expression		*P*-value
	Positive, *n* (%)	Negative, *n* (%)	
GC tissue (*n*=18)	10 (55.56)	8 (44.44)	0.04
Paracarcioma tissues (*n*=18)	4 (22.22)	14 (77.78)	

## Discussions

Invasive growth and distant metastasis are the two key features that characterize malignancy, which are also the primary reason for high mortality. Rapid proliferation and metastasis of tumors is facilitated by emergence of new blood vessels in the stroma [32]. Thus, inhibiting angiogenesis may be an effective strategy to inhibit cancer growth. The most significant feature of the *ADAMTS* family of enzymes are their diversity in thrombospondin type 1 (TSP1) motifs at the C-terminus. The TSP1 motifs are highly conserved and are an endogenous inhibitor of angiogenesis. It inhibits endothelial cell proliferation, induce endothelial cell apoptosis and anti-angiogenesis through its interaction with CD36 receptor [33].

In our study, GO enrichment analysis revealed that *ADAMTS* gene family is related to extracellular matrix/structure organization, extracellular matrix disassembly, and binding of cell adhesion molecules. Cell adhesion molecules mediate the interaction between cells or between the cells and matrix [34]. These adhesion molecules are synthesized and secreted by a variety of cells, and participate in the occurrence and metastasis of tumors [35]. In preventing tumor metastasis, extracellular matrix organization plays an important regulatory roles and its dissolution can promote tumor growth and metastasis. Previous data have suggested that the *ADAMTS* family of genes plays a vital role in the degradation of extracellular matrix [36]. Thus, the *ADAMTS* genes might be key anti-invasive molecules. The co-expression of the *ADAMTS*1–8 genes shows positive correlation with GC tumors. Nevertheless, data on the molecular expression and the role of *ADAMTS* set of genes in GC are still scant.

Several studies have revealed that *ADAMTS*1 is down-regulated in a variety of tumors [37–39], as well as in GC tumor tissue [40]. In our study, there was low expression of *ADAMTS*1 mRNA in GC tissues compared with those in adjacent tissues, *P*<0.001. Besides, the survival analysis also demonstrated that up-regulation of the *ADAMTS*1 is associated with poor survival time in GC patients (*P*=0.013), but not in multifactor analysis (*P*>0.05). Whereas the *ADAMTS*1 gene expression level correlates with OS in stomach cancer patients, it is not an independent prognostic biomarker for GC, thus more validation studies are required.

Compared with the normal tissues, *ADAMTS*2 expression in GC tumor cells and fibroblasts was significantly increased, and the up-regulation of the *ADAMTS*2 was associated with poor prognosis of GC patients [41]. Our data demonstrated that the expression of *ADAMTS*2 increases in GC, *P*<0.001, but the high expression did not affect mortality in GC (*P*>0.05).

It has also been observed that the increased expression of *ADAMTS*3 gene takes a major part in the development of myocardial infarction, osteoarthritis, and breast cancer [42]. Our study found the expression level of *ADAMTS*3 gene is not associated with the GC OS. Whereas *ADAMTS*4 expression was significantly up-regulated in invasive breast cancer tissues [43] and human glioma [44], our study showed that the expression of *ADAMTS*4 gene does not affect the OS of GC patients. Similarly, unlike previous studies [45–48], our findings shows that the expression profile of *ADAMTS*5 gene does not correlate with survival of GC patients. In addition, *ADAMTS*7 is involved in the migration and proliferation of smooth muscle and the development of atherosclerosis and restenosis [49]. Our current findings indicates the expression level of the *ADAMTS*7 gene is not related to OS in GC patients. Previous studies have shown that the *ADAMTS*8 expression is down-regulated in breast cancer, brain cancer, and non-small cell lung cancer [50–52]. Whereas our data showed down-regulation of the *ADAMTS*8 mRNA expression in GC, *P*<0.001, the low expression was not correlated with the risk of GC-specific mortality (*P*>0.05).

The up-regulation of *ADAMTS*6 is a molecular marker for poor prognosis in esophageal squamous cell carcinoma [53]. Besides, some researches have demonstrated that *ADAMTS*6 is dysregulated in breast cancer [54], prolactin tumors [55], and colorectal cancer [56]. In this study, we revealed that *ADAMTS*6 mRNA is overexpressed in GC tissues, *P*<0.001. *ADAMTS*6 expression is largely correlated with tumor stage, targeted molecular therapy, radical resection, radiation therapy, and histological grade of the GC. It was confirmed by immunohistochemistry and qRT-PCR that ADAMTS6 is up-regulated in GC, consistent with TCGA findings, which may be related to the prognosis of GC patients. Stratified analysis of the clinic pathological parameters such as age, gender, and TNM stage also showed that patients with the high expression of *ADAMTS*6 have reduced survival than those with low expression. Similarly, the multivariate survival and stratified analysis showed that the *ADAMTS*6 mRNA was up-regulated in GC patients and led to poor survival time. In addition, the *ADAMTS*6 gene was shown to promote the occurrence and development of stomach cancer. Our study revealed that *ADAMTS*6 is a tumor promoter, whose overexpression mediates occurrence, proliferation, invasion, or metastasis of stomach tumor, thus leading to a high mortality. Therefore, the *ADAMTS*6 gene may be a potential therapeutic target for GC.

The GSEA indicated that *ADAMTS*6 enriched cancer-related pathways, such as apoptosis, VEGF, KRAS, P53, JNK, CDH1, or TNF pathways, which may affect GC prognosis. Apoptosis plays a significant role in maintaining the stability of the internal environment. The balance between cell proliferation and apoptosis is pivotal to the stability of human internal environment, otherwise, any perturbation of this state might lead to tumorigenesis [57]. VEGF mediates angiogenesis, which has been considered as the strongest cytokine promoting tumor angiogenesis [58]. It has been shown that inhibition of the VEGF signaling pathway inhibits neovascularization, thus blocking the occurrence and metastasis of tumors [59]. KRAS is the most common mutation type in the RAS family of genes that affects the development of tumors [60,61]. Once KRAS mutates, it will lose the activity of GTP hydrolase, and thus continue to activate, promoting the uncontrolled cell proliferation and carcinogenesis. Besides, mutations in the p53 gene renders it ineffective in regulating cell growth, apoptosis, DNA repair and so on, causing cell transformation and cancer [62,63]. Deveci et al. demonstrated that the *P53* gene is associated with the occurrence and development of GC [64]. JNK signaling pathway plays a significant part in regulation of cell cycle, reproduction, apoptosis, and cell stress. Moreover, Yan et al., emphasized that inhibition of JNK signaling pathway may lead to apoptosis and metastasis of GC [65,66]. CDH1 gene mutation is a marker for poor GC prognosis [67–70]. TNF is involved in the inflammation and cellular immune response as well as tumor regulatory mechanisms [71–73]. The findings infer that *ADAMTS*6 may be involved in cancer-related pathways, including VEGF, KRAS, P53, JNK, CDH1, or TNF pathways, which play a crucial role in GC prognosis.

Our study has several limitations. Our study is dependent on data from a public database, thus the *ADAMTS* and survival analysis findings require further validation. Therefore, further experiment verification of the expression and function of *ADAMTS* is very necessary to improve the credibility of our current study. Besides, the underlying molecular mechanisms by which *ADAMTS*6 affects the occurrence and prognosis of GC needs further interrogation. In addition, because the information on TCGA database is incomplete, we were unable to clearly evaluate the relationship between the mRNA expression of the *ADAMTS* family of genes and protein expression. Subsequent studies need to reveal the biological mechanism of *ADAMTS*6 in the development and metastasis of GC from various aspects.

Despite these limitations, the present study is the first to investigate the correlation between *ADAMTS* mRNA expression and survive time in patients with stomach cancer. Log-rank test with Cox regression survival analysis and K–M survival analysis method were performed and found that *ADAMTS*6 expression level was largely correlated to GC patient clinical prognosis outcome. Thus, the prognostic relationship between *ADAMTS* family and GC was verified in the KM plotter database. Finally, GSEA found the differences in biological processes and related tumor pathways. Once these consequences are confirmed, we anticipate that *ADAMTS*6-targeted therapy drugs will be used in GC patients.

## Conclusions

Our research reveals that the up-regulation of *ADAMTS*6 is significantly associated with poor prognosis, and might be used as an independent predictive factor for GC. The potential mechanism of *ADAMTS*6 in GC prognosis was involved in cancer-related biologic processes and pathways, including apoptosis, VEGF, KRAS, P53, JNK, CDH1, or TNF pathways. Nevertheless, the potential mechanism of *ADAMTS*6 still need further verification and investigation.

## Data Availability

The data used to support the findings of the presewnt study are available from the corresponding author.

## Abbreviations

ADAMTS: a disintegrin and metalloproteinase with thrombospondin motifs
CDH1: cadherin
CI: confidence interval
FDR: false discovery rate
GC: gasctric cancer
GO: Gene Ontology
GSEA: Gene set enrichment analysis
HP: *Helicobacter pylori*
HR: hazard ratio
JNK: c-Jun N-terminal kinase
KM plotter: Kaplan–Meier plotter
KRAS: kirsten rat sarcoma viral oncogene
MMS: microsatellite stability
OS: overall survival
PPI: protein–protein interaction
ROC: receiver operating characteristic
TCGA: The Cancer Genome Atlas
TNF: tumor necrosis factor
TNM: T, tumour; N, node; M, metastasis
TSP1: thrombospondin type 1
